# Efficacy of a Multimodal Online Lifestyle Intervention for Depressive Symptoms and Quality of Life in Individuals With a History of Major Depressive Disorder

**DOI:** 10.7759/cureus.9061

**Published:** 2020-07-08

**Authors:** Robert D Abbott, Kyle Sherwin, Hannah Klopf, Holly J Mattingly, Kelly Brogan

**Affiliations:** 1 Integrative/Complementary Medicine, Resilient Roots, Charlottesville, USA; 2 Osteopathic Medicine, Midwestern University Arizona College of Osteopathic Medicine, Glendale, USA; 3 Osteopathic Medicine, Rocky Vista University College of Osteopathic Medicine, Denver, USA; 4 Counseling and Human Development, Lindsey Wilson College, Columbia, USA; 5 Psychiatry and Behavioral Sciences, Independent Researcher, Miami, USA

**Keywords:** depression, lifestyle, community, nutrition, quality of life, phq-9, sf-36, online learning, mental health, anxiety

## Abstract

Background

Major depressive disorder (MDD) is a complex bio-psycho-social syndrome that affects millions of individuals and is one of the leading causes of impaired quality of life (QOL). In addition to the symptoms of depression and low mood, many individuals with MDD also suffer from isolation without the sense of a supportive, surrounding community. Given the challenges of treating individuals with MDD, social isolation and a lack of communal connection, this randomized controlled trial was designed to determine the efficacy of a multimodal, online and community-based lifestyle intervention for improving depressive symptoms and QOL in individuals with a history of MDD.

Materials and methods

The study enrolled 71 female or male participants between the ages of 20 and 64 with a self-reported BMI between 18.4 and 34.9 kg/m^2 ^and a history of MDD. Individuals were randomized to either participate in a 44-day multimodal, online, community-based lifestyle intervention or placed on a wait list where they would complete the intervention at a later date. The multimodal intervention involved a self-directed learning program where individuals were guided to make lifestyle changes including adopting a whole-foods diet, increasing movement, and adopting stress management and mindfulness practices. All participants completed the 36-Item Short Form Health Survey (SF-36), the Cleveland Clinic Center for Functional Medicine's Medical Symptoms Questionnaire (MSQ), and the Patient Health Questionnaire-9 (PHQ-9) before and after the online program to assess health-related QOL, overall symptom burden, and depressive symptom burden, respectively.

Results

A total of 37 participants were randomized to participate in the multimodal intervention with 26 completing all three study questionnaires at both study time points; 34 participants were randomized to the wait list control group with 27 completing all three study questionnaires at both study time points. There were no clinically or statistically significant differences between the control group or the intervention group at baseline. The control group showed no clinically nor statistically significant changes in the MSQ, PHQ-9 or any of the eight subdomains of the SF-36 from the beginning to the end of the 10-week study period. When compared to the control group, the intervention group showed statistically and clinically significant improvements in median (M) scores of the SF-36 subdomains of vitality and mental health, and clinically but not statistically significant improvements in the subdomain of emotional role functioning. There were additional statistically and clinically significant improvements in the mean score of the MSQ and M scores of the PHQ-9 (treatment pre-intervention M = 10.5, inter-quartile range [IQR] = 14, to treatment post-intervention M = 5, IQR = 8.25; control pre-intervention M = 15, IQR = 8, to control post-intervention M = 13.5, IQR = 12.5).

Conclusions

Our randomized controlled study provides evidence for the role of a multimodal, online and community-based lifestyle intervention to improve depressive symptoms, QOL, and total symptom burden in individuals with a history of MDD. Given the growing challenges of effectively supporting individuals suffering with MDD, it appears critical to further explore the utilization of novel, multimodal and self-directed lifestyle interventions.

## Introduction

Major depressive disorder (MDD) is a complex bio-psycho-social syndrome with approximately 300 million individuals affected by at least one major depressive episode, globally [1]. In 2017, within the United States alone, it was estimated that 11 million adults had at least one major depressive episode resulting in severe impairment [1]. The WHO has declared depression to be the largest contributor to worldwide disability and the Institute of Medicine (Washington, DC) regards depression among the top 100 priorities for research [2,3].

Evidence from the controversial Sequenced Treatment Alternatives to Relieve Depression (STAR*D) trial indicated that despite medical management with first-line agents, depressive symptoms can persist in 67% of individuals after 14 weeks of pharmacologic treatment, with a further 30% remaining symptomatic after four, 12-week rounds of differing pharmacologic treatments [4,5]. When examining the most widely used anti-depressant drugs, it remains unclear which agents and which classes of medication should be considered first-line. Previous trials utilizing a variety of drug classes in both placebo-controlled and head-to-head studies have failed to show the superiority of any drug or elucidate the exact sequence that should be taken for those failing initial treatment [6,7]. While a 2018 systematic review and network meta-analysis examining the efficacy and tolerability of various anti-depressant agents indicated that all anti-depressants studied were more effective than placebo, there was much greater variability in tolerability and efficacy when certain anti-depressant medications were examined against each other in head-to-head trials [6]. In comparison, a 2019 systematic review and network meta-analysis that extended its search into both pharmacologic and non-pharmacologic treatment approaches for MDD found that while all medications studied were more effective than placebo, all non-pharmacologic treatments, with the exception of probiotic therapy, were also more effective than placebo with almost all of the examined medications having notably inferior tolerability profiles [8].

One of the primary theories surrounding the variability of pharmacologic responses involves the failure to adequately classify MDD into various subtypes based on severity, duration, disease course, or the presence of somatic symptoms [7,9]. Studies have shown that severe depression responds more favorably to medications than mild or moderate depression while treating mild depression with medications increases the risk of negative effects from over-treatment [8]. Given the potential for patients using anti-depressant medications to develop intolerable side effect profiles, there has been increasing interest in non-pharmacological strategies to treat and/or manage depression with or without the use of medications [8]. One such non-pharmacologic approach involves the positive modification of dietary and lifestyle habits.

Lifestyle medicine is a multimodal, whole systems, healthcare approach that promotes the modification of environmental, dietary, behavioral, and psychological habits in an effort to enhance physical and mental well-being [10]. More specifically, environmental modifications consist of tobacco cessation, avoidance of illicit substances, decreased consumption of alcohol, increased physical activity and increased time spent outdoors in natural green spaces. Dietary modifications focus on the exclusion of ultra-processed foods and the inclusion of more vegetables, fruits, nuts, seeds, lean proteins, seafood, and whole grains [10]. Behavioral and psychological modifications are promoted via sleep hygiene and stress management through mindfulness-based practices and meditation [10].

Unfortunately, well-conducted multimodal lifestyle medicine studies assessing the effectiveness and feasibility of interventions for MDD are lacking; however, two case reports and a recent systematic review were published with promising, although limited results, assessing the efficacy of online-based lifestyle intervention programs to improve lifestyle behaviors in depressed individuals [11-14].

Here, we attempt to study a specific multimodal, online, and community-based lifestyle medicine program to improve mood, cognitive functioning, behavioral symptoms, and quality of life (QOL) in individuals with a history of MDD. The program is a self-directed, but guided six-week program including education and promotion of tobacco cessation, moderate alcohol consumption, dietary modifications, increased physical activity, sleep hygiene, and stress reduction through meditation and mindfulness-based strategies.

## Materials and methods

Study design and measures

The study was designed as a two-arm, randomized controlled trial where participants were randomized into a treatment arm or wait list control group. Participants were recruited through various social media outlets and email correspondence to individuals who had previously expressed interest in the multimodal lifestyle intervention. Upon receiving correspondence from interested participants, a total of 147 individuals were enrolled and screened with inclusion and exclusion criteria by study investigators prior to study inclusion. The study, including recruitment, was conducted from January 2019 to March 2020.

Inclusion criteria consisted of English-speaking male or females between the ages of 20 and 64 with a diagnosis of MDD, and a BMI between 18.4 and 34.9 kg/m^2^. Exclusion criteria consisted of age or BMI greater or less than that described in inclusion criteria, no definitive diagnosis of MDD, pregnant, breastfeeding, six months postpartum, presence of other comorbidities including heart failure, liver failure, chronic or end-stage kidney disease, or being unable to complete a two-week washout period before the start of the trial. Following inclusion and exclusion criteria, 71 participants remained eligible for inclusion in the trial.

Participants performed a two-week washout period at the time of the screening visit. Here participants completed an informed consent, provided demographic information as well as current psychiatric medication use, past psychiatric medical history and current use of non-pharmacological substances. Study participants then completed study questionnaires that include a validated QOL survey, the 36-Item Short Form Health Survey (SF-36), the Cleveland Clinic Center for Functional Medicine’s Medical Symptoms Questionnaire (MSQ), and the Patient Health Questionnaire-9 (PHQ-9) [15-17].

Upon signing informed consent, study participants were randomized using a simple randomization scheme (random number generator) that indicated whether they would participate in the multimodal program after the completion of the two-week washout period or be placed on a wait list where they would be allowed to participate in the multimodal intervention at the end of the trial.

After completion of the washout period, the participants randomized to participate in the multimodal program were enrolled in a private, online community group and began the online program at an individualized pace for the next six weeks. The online program consisted of (a) educational materials on mental health; (b) dietary modifications promoting increased consumption of vegetables, fruits, nuts, seeds, lean proteins, and seafood and decreased consumption of ultra-processed foods; (c) sleep hygiene practices and stress management through mindfulness and meditation; (d) encouraging increased physical activity; and (e) increased social connectivity with other members in the private, online community.

As the study participants did not receive any personalized attention from the study team outside of the standardized educational materials in the six-week program, they were instructed to only contact the study team if they had questions about their participation in the study itself. At the end of the intervention or approximately 9-10 weeks after study participants completed their first set of study questionnaires, participants were asked to repeat the three study questionnaires over a two-week period.

The study was conducted in full accordance with Pearl IRB Research Policies and Procedures and all applicable Federal and State laws and regulations as well as the Good Clinical Practice: Consolidated Guidance approved by the International Conference on Harmonisation. Participants were informed that they were allowed to drop out of the study at any time. Adverse effects were monitored throughout the study and recorded.

Data collection, analysis, and outcomes

A per-protocol analysis was conducted using data from participants completing the study in its entirety. It was noted during initial statistical calculations that several subdomains of the SF-36 as well as the PHQ-9 failed the Shapiro-Wilk test or the D’Agostino and Pearson test for normality, and thus, all SF-36 data sets could not be assumed to be normally distributed. The MSQ, however, was normally distributed. The median and inter-quartile range (IQR) for dependent variables of the SF-36 and PHQ-9 were calculated utilizing the Wilcoxon signed rank tests between groups. The median and IQR for independent variables of the SF-36 and PHQ-9 were also calculated with the Mann-Whitney U test between groups. Paired t-tests for the MSQ were performed comparing dependent variables within control and intervention groups. Unpaired t-tests for the MSQ were performed comparing independent variables between control and intervention groups.

Mann-Whitney U tests were performed for age and BMI with results reported as medians and IQR for baseline demographics. To assess relevant psychiatric medical history, individuals were categorized and assigned a rank from 1 to 3 depending on the number of past psychiatric diagnoses. Individuals with a history of MDD without another psychiatric diagnosis were given a rank of 1. Individuals with a history of MDD plus another psychiatric diagnosis were given a rank of 2. Individuals with three or more psychiatric diagnosis were given a rank of 3. A Mann-Whitney U test was performed using these ranks in to compare the cumulative burden of psychiatric disease between the two study groups at baseline.

To assess current psychiatric medication use, individuals were categorized and assigned a rank from 0 to 3 depending on the number of psychiatric medications they were currently using. Individuals who were not using any psychiatric medications were given a rank of 0. Individuals who were taking only one psychiatric medication were given a rank of 1. Individuals taking two psychiatric medications were given a rank of 2. Individuals using three or more psychiatric medications were given a rank of 3. A Mann-Whitney U test was performed using these ranks in to compare the cumulative use of psychiatric medications between the two study groups at baseline.

To correct for error when performing statistical analyses for multiple associated hypotheses within the eight subdomains of the SF-36 questionnaire, balancing the risk of creating both Type I and Type II errors, the study authors utilized a false discovery rate control adjustment outlined by Glickman et al. with a maximum false discovery rate d = 0.05 for n = 8 statistical tests corresponding to the eight subdomains of the SF-36 [18].

As part of this adjustment, new thresholds for statistical significance were set for all four sets of calculations performed using the SF-36 data. As there were no p values < 0.05 comparing the dependent data of the control population pre- and post-intervention or the independent data of the control and treatment groups pre-intervention, the false discovery rate correction was only applicable for statistical analyses performed comparing the dependent data of the treatment population pre- and post-intervention and the independent data of the control and treatment groups post-intervention. 

The study's primary outcome was a significant change in SF-36 measures. The study's secondary outcomes consisted of changes in depressive symptoms as measured by the PHQ-9 and clinical symptom burden as measured by the MSQ. We hypothesized that, compared to the wait list control group, participants in the intervention group would see a clinically significant improvement in SF-36 and PHQ-9 scores. We hypothesized, despite the control group not receiving a therapeutic intervention, that the expectancy of participating in the intervention in the near future could result in clinical improvements for some of the measured scores of the control group from pre- to post-intervention. We did not have any a priori hypotheses regarding the magnitude of effect on SF-36, PHQ-9, or MSQ in either treatment arm.

## Results

A total of 71 participants met inclusion and exclusion criteria; 37 were randomized to the treatment group and 34 were randomized to the wait list control group.

In total, 27 of the 34 individuals in the wait list control group completed all three study questionnaires (SF-36, MSQ, and PHQ-9) before and after the conduction of the main study. One additional individual completed the PHQ-9 at both study testing time points, but failed to complete all aspects of the SF-36 and MSQ and thus could not be included in those analyses. Six participants did not complete questionnaires at the final study time point and were lost to follow-up.

A total of 26 of the 37 individuals in the treatment group completed all three study questionnaires (SF-36, MSQ, and PHQ-9) before and after the conduction of the trial. One participant completed the MSQ at both study time points, but failed to complete all aspects of the SF-36 or PHQ-9. Two participants could not complete the initial questionnaires during the washout period and eight participants did not complete questionnaires at the final study time point.

No adverse events were reported by any study participants.

Baseline demographic information including current psychiatric medical history, psychiatric diagnoses, and current psychiatric medication use by study participants is presented in Table 1. There were no statistically significant differences between the intervention group and the wait list control group at baseline for any of the demographic measures.

**Table 1:** Baseline demographics, psychiatric medical history, psychiatric medication use, and non-pharmacologic substance use MDD, major depressive disorder

	Intervention	Control	p
Age [median (IQR)]	38.0 (17)	41.0 (19.5)	0.285
BMI [median (IQR)]	22.5 (4.7)	25.0 (6.3)	0.145
Female (%)	85.2	89.3	0.705
Psychiatric medical history			0.748
MDD only diagnosis (%)	22.2	17.9	
2 Psychiatric diagnoses (%)	18.5	25.0	
3+ Psychiatric diagnoses (%)	59.3	57.1	
Current psychiatric medication use			0.0808
No medication (%)	33.3	57.1	
1 Psychiatric medication (%)	33.3	25.0	
2 Psychiatric medications (%)	22.2	10.7	
3+ Psychiatric medications (%)	11.1	7.2	
Current smokers (%)	0.0	3.6	>0.999
Minimum weekly alcohol use (%)	55.6	53.6	>0.999
Minimum weekly caffeine use (%)	66.7	75.0	0.562
Herbs and supplement use (%)	77.8	89.3	0.296

The pre- and post-intervention results and statistics of the wait list control group for the eight subdomains of the SF-36 are depicted in Table 2. Each subdomain of the SF-36 questionnaire is graded on a 100-point scale with higher values depicting a higher QOL within the corresponding subdomain. In examining the baseline measures for the wait list control group, it was noted that individuals did not report a clinically significant degree of impairment in the physical functioning, physical role functioning, or bodily pain subscales, but did report marked impairments in the emotional role functioning, vitality, mental health, and social role functioning subscales. There were no clinically nor statistically significant differences for any of the SF-36 subdomain measures post-intervention.

**Table 2:** SF-36 results and statistics pre- and post-intervention for the wait list control group SF-36, 36-Item Short Form Health Survey; pre, pre-intervention; post, post-intervention; IQR, inter-quartile range; N is the sample size.

	SF-36 physical functioning	SF-36 physical role functioning	SF-36 emotional role functioning	SF-36 vitality	SF-36 mental health	SF-36 social role functioning	SF-36 bodily pain	SF-36 general health
N	27	27	27	27	27	27	27	27
Median pre (IQR)	90 (15)	75 (75)	0 (33)	25 (30)	36 (20)	38 (25)	78 (43)	55 (30)
Median post (IQR)	90 (20)	50 (75)	0 (33)	20 (30)	44 (30)	38 (38)	68 (45)	55 (40)
Median of differences (IQR)	0 (10)	0 (50)	0 (0)	0 (20)	0 (12)	0 (38)	0 (25)	5 (15)
p	0.448	0.0523	0.824	0.883	0.682	0.300	0.471	0.233

Table 3 depicts the pre- and post-intervention results and statistics of the wait list control group for the PHQ-9. PHQ-9 scores range from 0 to 27 with scores of 5-9 indicating mild depression, 10-14 indicating moderate depression, 15-19 indicating moderately severe depression, and 20 and above indicating severe depression. The wait list control group began the study with a median PHQ-9 score of 15, IQR = 8, and finished the study with a median PHQ-9 score of 13.5, IQR = 12.5, resulting in no statistically significant changes.

**Table 3:** PHQ-9 results and statistics pre- and post-intervention for the wait list control group PHQ-9, Patient Health Questionnaire-9; pre, pre-intervention; post, post-intervention; IQR, inter-quartile range; N is the sample size.

	N	PHQ-9
Median pre (IQR)	28	15 (8)
Median post (IQR)	28	13.5 (12.5)
Median of differences (IQR)	28	-1 (4.75)
p	28	0.3991

Table 4 depicts the pre- and post-intervention results and statistics of the wait list control group for the MSQ. The MSQ cumulative score is derived from the summation of 15 "system" scores derived from the summation of three to eight components of each "system" score. Each component of the system score is ranked 0-4 based on the frequency and severity of a given symptom over the past month, with a score of 0 indicating the lack of a given symptom, 1 indicating the occasional, but not severe presence of the symptom, 2 indicating the occasional and severe presence of the symptom, 3 indicating the frequent, but not severe presence of the symptom, and 4 indicating the frequent and severe presence of the symptom. Scores can range from 0 to 284 with higher scores indicating the presence of a higher symptom burden based on both frequency and severity of symptoms. The wait list control group began the study with a mean MSQ score of 76, SD = 30, and finished the study with a mean score of 74, SD = 30, resulting in no statistically nor clinically significant changes.

**Table 4:** MSQ results and statistics pre- and post-intervention for the wait list control group MSQ, Medical Symptoms Questionnaire; pre, pre-intervention; post, post-intervention

	N	MSQ
Mean pre (SD)	27	76 (30)
Mean post (SD)	27	74 (30)
Mean of differences (SD)	27	-2 (21)
p	27	0.667

Pre- and post-intervention results and statistics of the intervention group for the eight subdomains of the SF-36 are depicted in Table 5. Clinically and statistically significant improvements occurred in the vitality, mental health, bodily pain, and general health subdomain measures of the SF-36 from baseline. Additionally, there was a clinically, but not statistically significant change in the emotional role functioning subscale and a statistically, but not clinically significant change in the physical functioning subscale.

**Table 5:** SF-36 results and statistics pre- and post-intervention for treatment SF-36, 36-Item Short Form Health Survey; pre, pre-intervention; post, post-intervention; IQR, inter-quartile range; N is the sample size. *Clinically and statistically significant, **statistically but not clinically significant, ***clinically but not statistically significant.

	SF-36 physical functioning	SF-36 physical role functioning	SF-36 emotional role functioning	SF-36 vitality	SF-36 mental health	SF-36 social role functioning	SF-36 bodily pain	SF-36 general health
N	26	26	26	26	26	26	26	26
Median pre (IQR)	90 (20)	50 (100)	0 (67)	28 (35)	42 (26)	50 (41)	58 (45)	48 (36)
Median post (IQR)	90 (20)	63 (100)	50 (71)	45 (25)	64 (28)	56 (38)	80 (23)	65 (21)
Median of differences (IQR)	5 (10)	0 (50)	0 (70)	20 (30)	22 (32)	13 (31)	13 (35)	10 (26)
p	0.0243**	0.971	0.122***	<0.0001*	0.0006*	0.0607	0.0034*	0.0028*

Within-group PHQ-9 results for the treatment group revealed statistically and clinically significant decreases in depressive symptoms (Table 6).

**Table 6:** PHQ-9 results and statistics pre- and post-intervention for the treatment group PHQ-9, Patient Health Questionnaire-9; pre, pre-intervention; post, post-intervention; IQR, inter-quartile range; N is the sample size.

	N	PHQ-9
Median pre (IQR)	26	10.5 (14)
Median post (IQR)	26	5 (8.25)
Median of differences (IQR)	26	-3 (8.25)
p	26	<0.0001

Within-group MSQ results for the treatment group were also statistically and clinically significant with decreases in the overall symptom burden (Table 7).

**Table 7:** MSQ results and statistics pre- and post-intervention for the treatment group MSQ, Medical Symptoms Questionnaire; pre, pre-intervention; post, post-intervention

	N	MSQ
Mean pre (SD)	27	79 (29)
Mean post (SD)	27	42 (29)
Mean of differences (SD)	27	-36 (31)
p	27	<0.0001

There were no statistically significant differences between the two groups in any SF-36 domain at baseline; however, statistically and clinically significant differences for the mental health and vitality subdomains were seen post-intervention (Table 8). Clinically significant changes in the emotional role functioning domain did not remain statistically significant after correcting for multiple hypothesis testing. There was additionally a clinically, but not statistically meaningful improvement in the bodily pain subdomain for the treatment group as compared to a worsening in the bodily pain score for the control group.

**Table 8:** SF-36 results and statistics pre- and post-intervention for the wait list control group compared to the treatment group SF-36, 36-Item Short Form Health Survey; pre, pre-intervention; post, post-intervention; IQR, inter-quartile range; N is the sample size. *Statistically significant, **did not meet corrected statistical significance after false detection rate correction.

	N	SF-36 physical functioning	SF-36 physical role functioning	SF-36 emotional role functioning	SF-36 vitality	SF-36 mental health	SF-36 social role functioning	SF-36 bodily pain	SF-36 general health
Median control pre (IQR)	27	90 (15)	75 (75)	0 (33)	25 (30)	36 (20)	38 (25)	78 (43)	55 (30)
Median treatment pre (IQR)	26	90 (20)	50 (100)	0 (67)	28 (35)	42 (26)	50 (41)	58 (45)	48 (36)
p		0.388	0.610	0.425	0.544	0.214	0.214	0.182	0.700
Median control post (IQR)	27	90 (20)	50 (75)	0 (33)	20 (30)	44 (32)	38 (38)	68 (45)	55 (40)
Median treatment post (IQR)	26	90 (20)	63 (100)	50 (71)	45 (25)	64 (28)	56 (38)	80 (23)	65 (21)
p		0.296	0.302	0.0215**	0.0066*	<0.0001*	0.114	0.0573	0.196

**Figure 1:**
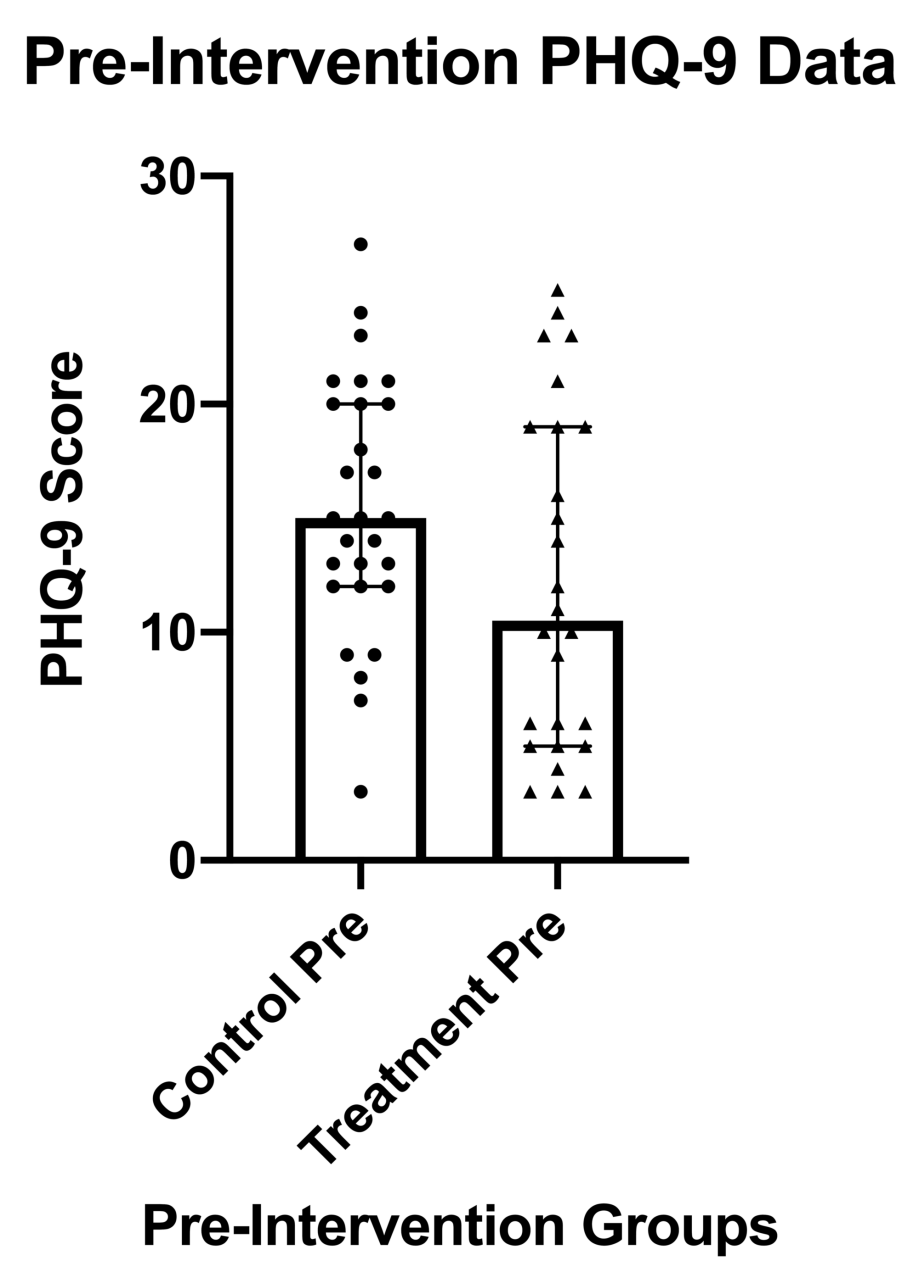
PHQ-9 results and statistics pre-intervention for the treatment group compared to the control group PHQ-9, Patient Health Questionnaire-9; pre, pre-intervention; IQR, inter-quartile range; error bars indicate the IQR and bold rectangles indicate the median score.

Figure 1 comparatively depicts the baseline median PHQ-9 scores between groups. Despite the intervention group having a lower baseline median PHQ-9 score, these differences were not statistically significant (p=0.0762).

**Figure 2:**
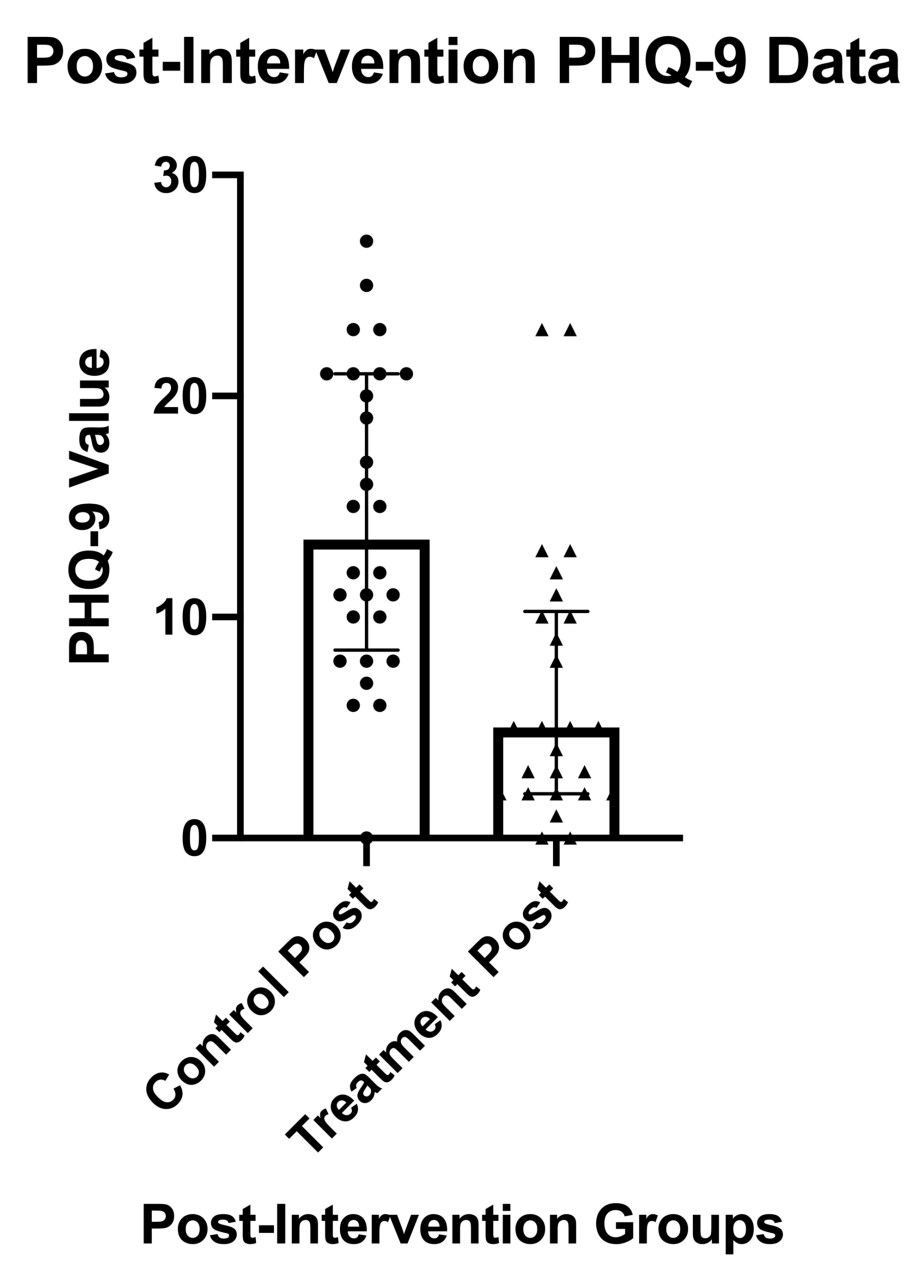
PHQ-9 results and statistics post-intervention for the treatment group compared to the control group PHQ-9, Patient Health Questionnaire-9; post, post-intervention; IQR, inter-quartile range; error bars indicate the IQR and bold rectangles indicate the median score.

Figure 2 comparatively depicts the post-intervention median PHQ-9 scores between groups. The median post-intervention PHQ-9 score in the control group was 13.5, IQR = 12.5, vs 5.0, IQR = 8.25 (p<0.0001) in the treatment group, which resulted in a clinically and statistically significant difference.

**Figure 3:**
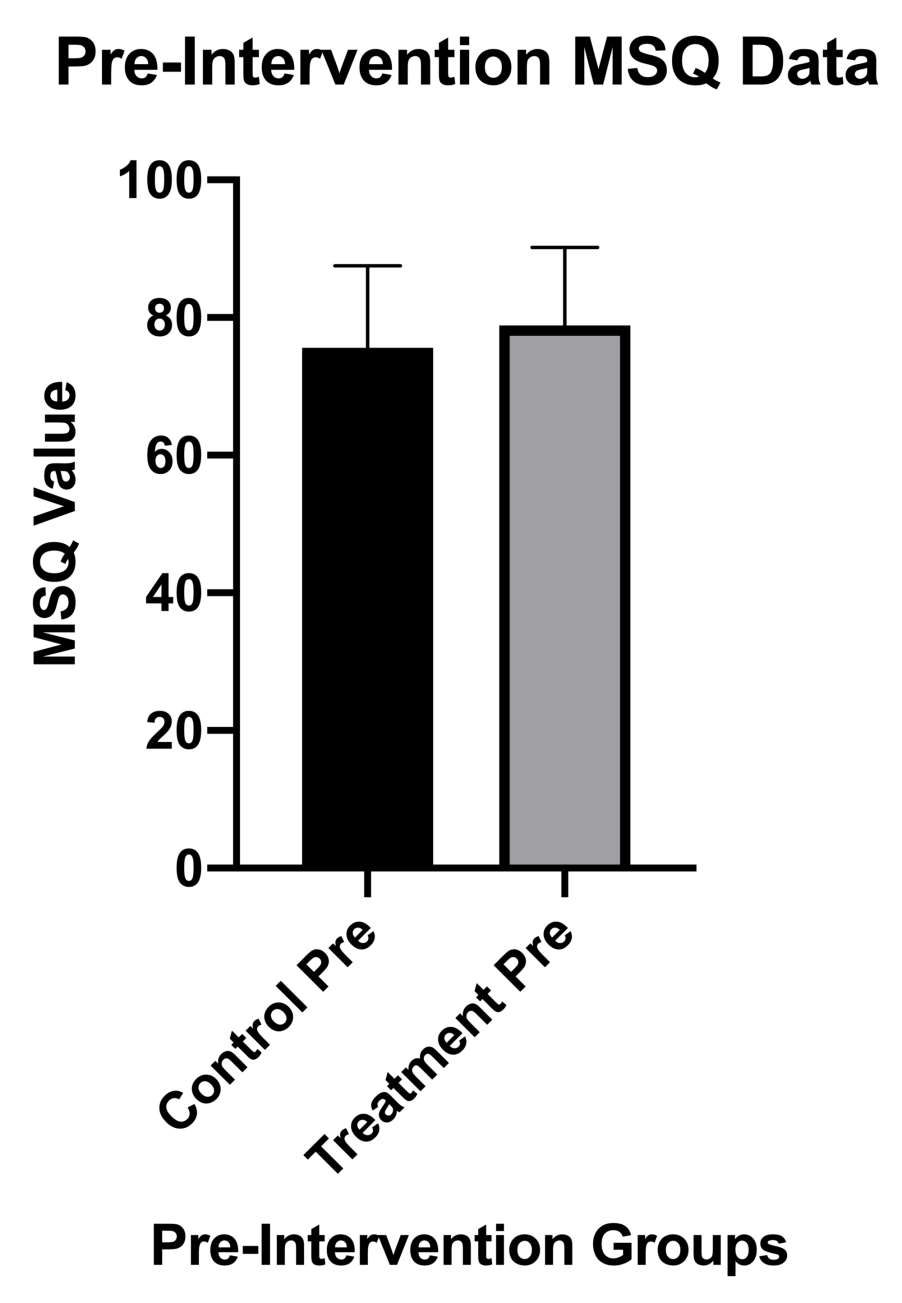
MSQ results and statistics pre-intervention for the treatment group compared to the control group MSQ, Medical Symptoms Questionnaire; pre, pre-intervention; error bars indicate SD.

Figure 3 comparatively depicts the baseline MSQ scores between groups with no statistically significant differences between groups (p=0.685).

**Figure 4:**
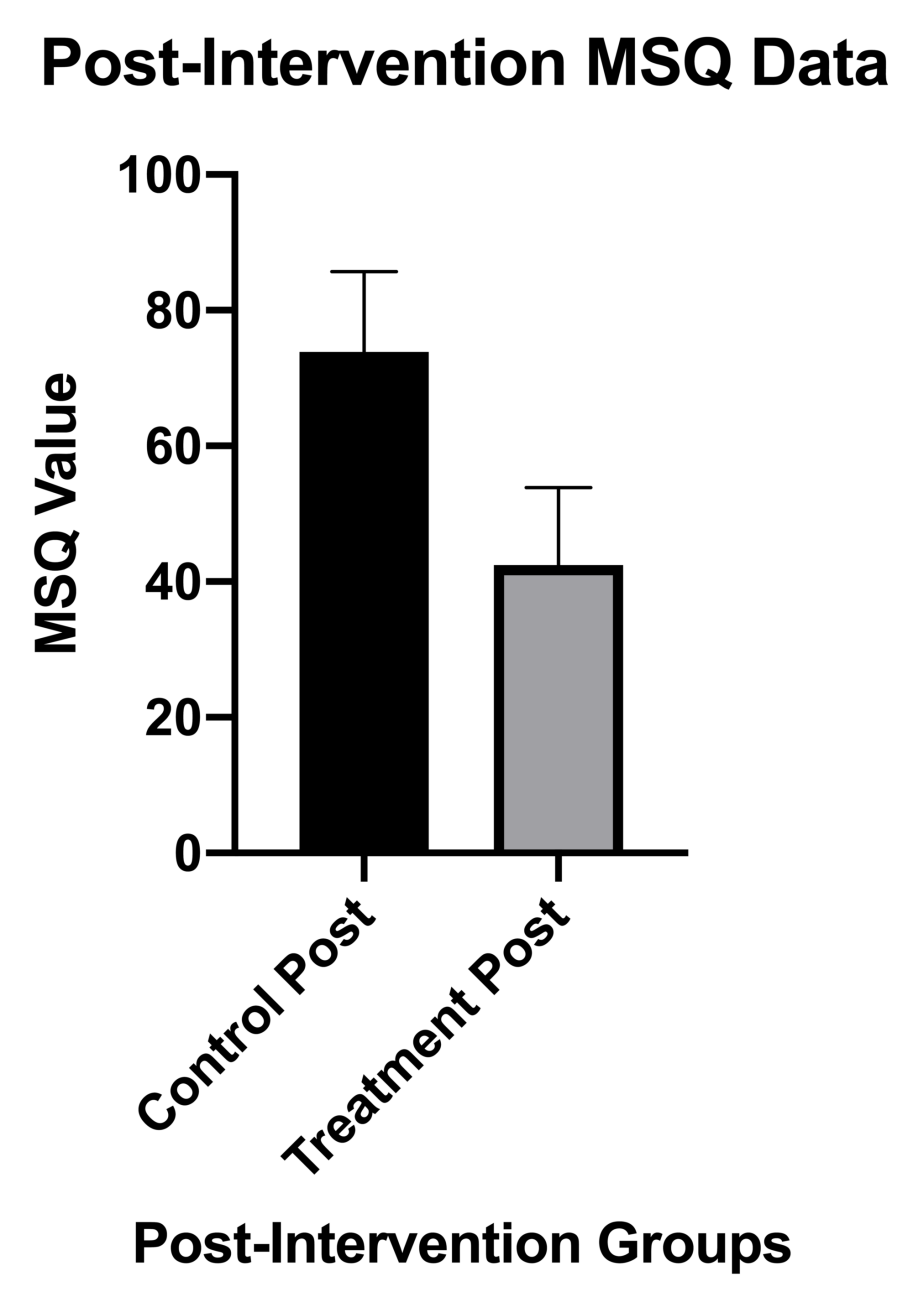
MSQ results and statistics post-intervention for the treatment group compared to the control group MSQ, Medical Symptoms Questionnaire; post, post-intervention; error bars indicate SD.

Figure 4 comparatively depicts the post-intervention MSQ scores between groups, which revealed statistically and clinically significant differences between the two groups post-intervention (p=0.0003).

## Discussion

We aimed to investigate the delivery of lifestyle medicine practices through an online, multimodal program to reduce symptoms of depression and improve QOL in patients with MDD. We demonstrated preliminary efficacy via statistically and clinically significant improvements in SF-36, PHQ-9, and MSQ scores. Individuals completing the multimodal online program showed a 52% decrease in the median PHQ-9 score resulting in clinically significant improvements in moderate depression, whereas these improvements were not seen in the control group. Similarly, a 46% reduction in total symptom burden as measured by the MSQ was seen in the intervention group, with a lack of improvement seen in controls. These same trends were also noted for several subscales of the SF-36 indicating overall health-related QOL improvements in the intervention, but not the control group.

When examining the past psychiatric medical history, we observed significant comorbidity with the majority of individuals in both study groups reporting two or more psychiatric diagnoses in addition to a history of MDD. When examining current psychiatric medication use, we observed a slightly higher amount of individuals in the control group reporting no current use of psychiatric medications as compared to the intervention group. The number of psychiatric medications used per individual, however, was statistically similar between the two groups.

As there are multiple confounders present in the multifactorial lifestyle intervention, we can only speculate as to which lifestyle modification(s) was responsible for eliciting the observed treatment effects. Multiple studies have suggested that a diet low in ultra-processed foods and high in fruits and vegetables is protective against depression, with depressed patients consistently showing lower intakes of fruits, vegetables, fish, folate, calcium, and magnesium [19-21]. One small study found that vitamin D supplementation in conjunction with standard of care significantly decreased depressive symptoms [22]. A systematic review on the effect of dietary polyphenols on depression concluded that higher intake of polyphenols, commonly seen in the Mediterranean diet, is associated with a lower prevalence of depression [23]. While we cannot determine the specific treatment effect of the dietary guidance provided in the multimodal program, our study findings appear consistent with previously published findings associating dietary intake with MDD.

Lopresti et al. estimated that approximately 90% of MDD patients have sleep disturbances, with those suffering from insomnia having worse responses to treatment [24]. The multimodal program's inclusion of sleep hygiene education was, therefore, likely beneficial to individuals in the treatment group suffering from insomnia and disturbed sleep patterns. Extending the discussion into mindfulness-based practices, a meta-analysis of several high-quality trials suggested that mindfulness-based cognitive therapy (MBCT) was an effective and feasible therapy for adults with at least three or more previous episodes of MDD [25]. Further research into the potential mechanisms behind the benefits of mindfulness-based therapies indicated that emotional regulation is a critical mediator of meditation practices [26]. Furthermore, meditation practices that seek to minimize worry and rumination appear even more essential for individuals at risk of depression [26]. While the multimodal program employed in this study did not utilize a regimented protocol such as MBCT or mindfulness-based stress reduction (MBSR), the meditation and mindfulness practices provided to participants were similar in nature to the types of practices included in MBCT and MBSR.

In light of these research findings, when examined collectively, it is very likely that individuals completing the multimodal program achieved synergistic benefits from the inclusion of multiple treatment elements. It is speculated as well that individuals likely achieved greater benefit from specific treatment elements depending on their baseline lifestyle habits. Given the great concern for excessive study burden and the limitations of the virtually based study team, specific measures of sleep hygiene, exercise pattern, and dietary adherence were not utilized, and as such, we can only speculate as to the differential treatment effects of individual therapies.

While the focus of this discussion thus far has entertained the nature of the education provided to the participants, it is also critical to address the method of delivery and implementation of this education. The multimodal program allowed participants to implement changes in a self-directed manner. We hypothesize that the self-directed structure likely contributed to more successful outcomes increasing self-efficacy and autonomy [27]. In addition, participants were allowed and encouraged to interact with other people who were simultaneously completing the same multimodal program on their own accord. The social interaction occurring between study participants with other individuals making similar self-directed lifestyle changes could have helped patients with MDD in two ways. First, the social interaction could have assisted patients by increasing adherence to the program via greater social support and external accountability. Second, the social interactions intrinsically could have improved the symptoms by increasing positive social interactions qualitatively and quantitatively. The ability to interact with others may have played a definitive role in ensuring success of the study participants as the quality of social relationships appears to be a major risk factor for MDD [28]. As there was no feasible technology to track the nature or frequency of these social interactions between our study participants, further research should seek to quantify the level of social engagement between participants as part of community-based lifestyle interventions.

Many of the challenges faced by both clinical providers and patients when seeking to implement lifestyle changes involve the limited time and resources of the provider and the limited capacity for adherence by the patient. The use of online and community-based interventions could be instrumental for practitioners treating MDD since the implementation of such interventions requires little time from the physician. Such online interventions could help relieve the burden on the physician to support the patient in making these changes during time-restricted office visits.The utilization of online, community-based treatments could also give the physician and patient flexibility during the treatment period to schedule follow-up appointments assessing progress and adherence to the program. Allowing the physician and patient to engage in dynamic shared decision-making, we would likely see an even greater promotion of patient autonomy alongside clinical improvements. As the delivery of clinical services transitions to a greater reliance on telehealth and online platforms, web-based interventions will only become more critical to address the growing burden of depression.

The limitations of this study include a 21% failed completion rate in the control group and a 30% failed completion rate in the intervention group. This difference in completion rates may be attributable to the fact that the control group had to complete the second set of study surveys to gain access to the multimodal program while the intervention group did not have any incentive to complete the second set of questionnaires after finishing the online program. It is also possible that both groups experienced questionnaire fatigue contributing to lower than expected survey completion rates. Future studies should employ better methods for ensuring participant completion of study questionnaires at all time points.

In addition to the higher than expected failed completion rates, the study is also limited by selection bias since people who were selected to participate in the study were recruited primarily from individuals who had previously shown interest in the program or were aware of the education provided in the program from previously published books and online educational material. As the study primarily focused on non-smoking, middle-aged females, we must be cautious to generalize the findings of this relatively small study to a larger population. While previous research into the gender differences of depressive illness has revealed significantly higher levels of depression in females than males, our study's number of female participants was still higher than population-based current estimates [29]. In addition, our study had only one participant who acknowledged active tobacco use. Previous research has suggested significant comorbidity between depression and smoking, with depressed individuals being more likely to smoke when compared to non-depressed individuals [30].

Given the lack of consensus for the relevance of any objective measure to adequately indicate an individual's level of depression and impaired QOL, we utilized study questionnaires focused on subjective data that can vary significantly with the greatest impacts seen in relation to an acute worsening of mental or physical health. While the PHQ-9 and SF-36 questionnaires are well-validated measures to assess depressive symptoms and health-related QOL, respectively, the MSQ (questionnaire) used in this study is yet to be validated as a reliable questionnaire for quantifying the collective symptom burden.

## Conclusions

Our randomized controlled trial provides evidence for the role of a multimodal lifestyle intervention to improve depressive symptoms, QOL, and total symptom burden in individuals with a history of MDD. While further research utilizing similar multimodal, self-directed lifestyle interventions that leverage the power of communal engagement and virtual education should be undertaken with larger and more diverse patient populations, our study adds to the existing literature that lifestyle and community-based medicine interventions are viable and low-risk therapies can aid in the management of MDD.
